# Development and initial validation of an academic entitlement scale in Egyptian college students

**DOI:** 10.1186/s40359-025-03740-7

**Published:** 2025-12-05

**Authors:** Ashraf Ragab Ibrahim, Mohamed Ali Nemt-allah

**Affiliations:** Educational Psychology and Statistics Department, Faculty of Education, Al-Azhar University, Tafhna Al-Ashraf, Dakahlia, Egypt

**Keywords:** Academic entitlement, Egyptian higher education, Psychometric properties, Reliability, Validity

## Abstract

**Background:**

Academic entitlement represents students' expectations of academic success without personal responsibility. Despite growing research on academic entitlement in Western contexts, validated measurement tools for non-Western educational settings remain limited. This study aimed to develop and validate an academic entitlement Scale specifically designed for use within Egyptian higher education.

**Methods:**

A quantitative methodology was employed with 1,327 undergraduate students from Al-Azhar University's Faculty of Education. The study utilized a 43-item Academic Entitlement Scale, which underwent expert review and translation. Data analysis included exploratory factor analysis (EFA) with 728 students (370 males, 358 females), confirmatory factor analysis (CFA) with 599 students (161 males, 438 females), reliability assessment through multiple methods (Cronbach's alpha, McDonald's omega, test–retest reliability), and validity examination through correlational analyses.

**Results:**

EFA revealed a five-factor structure accounting for 42.8% of total variance, initially resulting in a 34-item scale. Following CFA model refinement, a final 28-item scale emerged. The factors identified were: Reward for Minimal Effort, Control Over Learning Environment, Consumer Mindset, Entitled Expectations, and Externalized Responsibility. CFA confirmed this structure with satisfactory fit indices (CFI = 0.918, RMSEA = 0.045, CMIN/DF = 2.207). The scale demonstrated good convergent validity (AVE = 0.574) and composite reliability (CR = 0.869). Inter-factor correlations ranged from 0.366 to 0.551, supporting discriminant validity while confirming their collective representation of the broader academic entitlement construct. Reliability coefficients were robust across multiple metrics (ω = 0.714–0.842; α = 0.710–0.841), with the total scale showing excellent reliability (ω = 0.902). Test–retest reliability over 17 days demonstrated acceptable to good temporal stability (*r* = 0.561–0.870).

**Conclusions:**

The Academic Entitlement Scale demonstrates satisfactory psychometric properties within Egyptian higher education, capturing the multidimensional nature of academic entitlement in this cultural context. The emergence of Control Over Learning Environment and Consumer Mindset as distinct factors suggests unique cultural considerations in Egyptian students' academic expectations. This validated scale provides a valuable tool for researchers and educators to assess and address entitlement behaviors in Arab university settings, contributing to a more globally inclusive understanding of academic entitlement.

## Background

Academic entitlement represents a growing concern in contemporary higher education, characterized by students' expectations of academic success without taking personal responsibility for achievement [1, 2]. This phenomenon has emerged as a significant challenge that threatens the integrity of educational processes and undermines the fundamental principles of academic merit and effort-based learning. The concept originated from broader research on generalized entitlement and narcissism, subsequently adapted specifically to educational contexts to address the unique manifestations of entitlement behaviors within academic settings [3].

The theoretical foundation of academic entitlement encompasses multiple dimensions that reflect the complexity of entitled academic behaviors. Research has identified various conceptualizations of this construct, including its relationship to external locus of control [5], entitled expectations [6], and externalized responsibility [7]. These conceptual frameworks highlight how academic entitlement differs fundamentally from general psychological entitlement through its domain-specific focus on academic contexts and its particular emphasis on the expectation of success without corresponding effort.

Academic entitlement manifests distinctively from general psychological entitlement through its specific characteristics of expecting academic success without effort and consistently externalizing responsibility for academic outcomes [8, 9]. Students exhibiting high levels of academic entitlement typically demonstrate narcissistic tendencies alongside unrealistic grade-related expectations [10]. The construct shows meaningful associations with reduced academic motivation and self-efficacy [5], decreased honesty-humility [6], and psychological resilience [11]. Furthermore, academic entitlement has been empirically linked to increased levels of depression and anxiety [12], higher rates of study absences [13], reduced moral intelligence [14], and diminished life satisfaction [15], demonstrating its far-reaching impact on student well-being and academic engagement.

### Problem situation and educational impact

The rise of academic entitlement in higher education institutions presents multifaceted challenges that extend beyond individual student performance to affect entire educational ecosystems. Students with elevated academic entitlement levels consistently exhibit concerning behavioral patterns that disrupt traditional academic environments. These behaviors include reduced honesty-humility, heightened narcissism, and the presence of Dark Triad personality traits [6, 16, 17]. Such students frequently display diminished intrinsic motivation coupled with an external locus of control [5, 18], consistently attributing their academic outcomes to external factors rather than acknowledging the role of personal effort and preparation [19].

The manifestation of academic entitlement creates a cascade of problems within educational institutions. Students with high academic entitlement levels are significantly more prone to engaging in academic dishonesty while simultaneously maintaining unrealistic expectations for special accommodations and preferential treatment [20]. These individuals often experience lower self-esteem and reduced self-efficacy [21], creating a paradoxical situation where their entitled attitudes mask underlying feelings of inadequacy and insecurity. The resulting behavioral patterns contribute to grade inflation pressures, increased faculty burnout, and elevated levels of student incivility within academic settings [22, 23].

Recent literature has documented several specific mechanisms through which academic entitlement contributes to grade inflation pressures and faculty burnout. Student consumerism has emerged as a primary driver, with students increasingly viewing education as a commodity and expecting higher grades as part of their educational "purchase" [24]. This shift creates significant pressure on faculty to inflate grades to maintain student satisfaction. Additionally, Student Evaluations of Teaching (SETs) have been identified as a critical factor, as faculty often feel compelled to award higher grades to secure favorable evaluations that directly impact their promotion, tenure, and job security [25, 26]. Adjunct faculty face particular vulnerability to these pressures, reporting greater likelihood of grade inflation due to concerns over contract renewal and lack of institutional support compared to tenured faculty [27, 28]. Furthermore, academic entitlement has been linked to increased student incivility, which directly contributes to faculty strain and burnout through emotional exhaustion from repeated grade disputes and the constant need to justify academic standards [29].

Quantitative research has established robust associations between academic entitlement and student incivility, though specific prevalence rates remain limited in the literature. Studies demonstrate that increased academic entitlement among undergraduates correlates significantly with higher acceptance of plagiarism, academic dishonesty, and classroom incivility [30]. Latent means modeling reveals that students classified as "uncivil/noncompliant" score significantly higher on academic entitlement measures compared to their "civil/compliant" peers [31]. Higher student academic entitlement has been significantly related to instructor-reported uncivil behaviors, with these behaviors fully mediating the relationship between entitlement and instructor strain [29]. Faculty surveys indicate that 30% to 71% perceive incivility as a moderate or serious problem in educational environments, with entitlement attitudes frequently cited as contributing factors [32].

The impact of academic entitlement on student-faculty relationships represents a particularly concerning aspect of this phenomenon. Students with high entitlement levels frequently engage in distributive bargaining and conflict with faculty members [33], displaying uncivil behaviors that create significant strain on educators and compromise the quality of educational interactions [29]. These students maintain unrealistic expectations that ultimately compromise educational quality and undermine the learning environment for all students [34]. Faculty members, particularly those in lower academic ranks, report perceiving higher levels of student entitlement, which directly affects their job-related well-being and professional satisfaction [35, 36].

### Limitations of existing measures

Several validated instruments have been developed to measure academic entitlement, each with distinct theoretical orientations and psychometric properties. The Academic Entitlement Scale developed by Jackson et al. [4] represents one of the foundational measures in this area, identifying seven distinct dimensions of academic entitlement through comprehensive factor analysis. The Academic Entitlement Questionnaire (AEQ) [37, 38] offers an alternative approach to measurement, while culturally adapted versions have been developed for specific populations, including Turkish university students [39].

Despite these existing instruments, significant limitations constrain their applicability across diverse cultural and educational contexts. Most existing studies have concentrated on Western student populations, particularly those in North American and European institutions, raising important questions about the cross-cultural validity and applicability of these scales [6, 37]. The cultural specificity of entitled behaviors and expectations suggests that direct translation and application of existing instruments may not adequately capture the nuances of academic entitlement as it manifests in different educational systems and cultural contexts.

The Egyptian higher education landscape exhibits distinct features that may not be fully reflected in instruments originally developed within Western contexts. Cultural norms, pedagogical traditions, and the dynamics of student-faculty interactions in Egyptian universities are likely to shape both the expression and interpretation of academic entitlement in unique ways. To the best of the researchers’ knowledge, a systematically validated academic entitlement scale specifically designed for Egyptian university students has not yet been introduced, despite growing recognition of entitlement-related academic challenges in the region.

### Study objectives

Given the identified limitations in existing measures and the unique characteristics of the Egyptian higher education context, this study aims to develop and validate a culturally appropriate Academic Entitlement Scale for Egyptian university students. The specific objectives are to: (1) investigate the underlying factor structure of academic entitlement through EFA within the Egyptian context, (2) validate the identified factor structure using CFA with an independent, (3) assess measurement reliability through multiple approaches including internal consistency measures (McDonald's omega, Cronbach's alpha), test–retest stability assessment, and composite reliability evaluation, (4) evaluate overall model fit using comprehensive fit indices to ensure adequate representation of the construct, and (5) examine inter-factor relationships to understand the dimensional associations within the Egyptian context.

These objectives collectively address the critical need for a psychometrically robust instrument to measure academic entitlement within Egyptian higher education, filling an important gap in cross-cultural assessment tools for this significant educational construct. The study contributes to the broader understanding of academic entitlement across cultural contexts while providing educators and researchers with a validated tool for investigating this phenomenon within the Egyptian university system.

## Method

### Research design

The study employed a quantitative research methodology to examine the psychometric properties of the Academic Entitlement Scale when applied to students in Al-Azhar University. The research specifically focused on investigating the scale's validity and reliability within the cultural and educational context of Egyptian universities. The study was conducted during the academic year 2023–2024 at the Faculty of Education for Boys in Tafahna Al-Ashraf, Dakahlia Governorate, and the Faculty of Education for Girls in Cairo.

### Participants and sampling

Participants were selected through convenience sampling from multiple academic departments across two faculties due to limited access to a comprehensive sampling frame and the study's exploratory nature. The total sample comprised 1,327 undergraduate students who provided complete and valid responses across both phases of the study. For the EFA phase, 728 students participated (370 males, 50.8%; 358 females, 49.2%), with ages ranging from 18 to 24 years (M = 20.00, SD = 1.44). The CFA phase included 599 students (161 males, 26.9%; 438 females, 73.1%), with ages ranging from 17 to 23 years (M = 20.76, SD = 1.00).

Participants were drawn from six academic specializations within the education faculties: Arabic Language Education, French Language Education, English Language Education, Special Education, Educational Technology, and Geography Education. The sample represented all undergraduate academic levels, from first-year through fourth-year students, ensuring adequate representation across different stages of university education. Demographic analysis revealed that third-year students constituted the largest proportion of the EFA sample (39.7%, *n* = 289), while Special Education majors represented the most substantial academic specialization (33.2%, *n* = 242). In terms of residential background, urban students comprised 75.9% (*n* = 553) of the EFA sample, with rural students accounting for 24.1% (*n* = 175). The CFA sample demonstrated a different demographic distribution, with third-year students representing 53.1% (*n* = 318) and urban students comprising 60.1% (*n* = 360). Table 1 presents the comprehensive demographic and academic distribution of participants across both study phases.

**Table 1:** Demographic and academic characteristics of study participants across EFA and CFA Phases

Category	Subgroup	EFA Sample (*N* = 728)			CFA Sample (*N* = 599)		
		**Male (%)**	**Female(%)**	**Total**	**Male (%)**	**Female(%)**	**Total**
Academic Year	First Year	90(50.6%)	88(49.4%)	178	52(58.4%)	37(41.6%)	89
	Second Year	30(52.6%)	27(47.4%)	57	24(28.2%)	61(71.8%)	85
	Third Year	150(51.9%)	139(48.1%)	289	126(39.6%)	192(60.4%)	318
	Fourth Year	100(49.0%)	104(51.0%)	204	37(34.6%)	70(65.4%)	107
Major	Arabic	40(51.3%)	38(48.7%)	78	14(35.9%)	25(64.1%)	39
	French	25(45.5%)	30(54.5%)	55	17(38.6%)	27(61.4%)	44
	English	85(50.6%)	83(49.4%)	168	50(32.7%)	103(67.3%)	153
	Special Ed	120(49.6%)	122(50.4%)	242	86(39.1%)	134(60.9%)	220
	Educational Technology	61(100%)	0(0%)	61	37(100%)	0(0%)	37
	Geography	48(38.7%)	76(61.3%)	124	35(33.0%)	71(67.0%)	106
Residence	Rural	95(54.3%)	80(45.7%)	175	66(27.6%)	173(72.4%)	239
	Urban	275(49.7%)	278(50.3%)	553	95(26.4%)	265(73.6%)	360

Table 1 provides a comprehensive overview of the study's sample, illustrating the diverse representation across academic years, departments, and residential backgrounds. The table reveals that third-year students comprised the largest cohort (39.7%), with Special Education being the most represented academic department (33.2%). The sample had a relatively balanced gender distribution with 50.8% male and 49.2% female participants.

### Instrument

The primary research instrument was an original Academic Entitlement Scale developed specifically for this study, initially consisting of 49 items designed to measure various aspects of academic entitlement among Egyptian university students. The scale employed a 5-point Likert-type response format ranging from 1 (strongly disagree) to 5 (strongly agree).

To ensure content validity and cultural appropriateness, the scale was reviewed by twelve specialized psychology experts who evaluated items across six criteria including appropriateness of components, item relevance, scale suitability, scoring appropriateness, clarity of formulation, and population suitability. Quantitative content validity was assessed using Lawshe's Content Validity Ratio (CVR), with expert agreement ranging from 92 to 100% across all criteria. The overall Content Validity Index (CVI) was 0.726, exceeding the minimum threshold of 0.58 for twelve experts, indicating acceptable expert consensus. Based on expert recommendations and quantitative analysis, six items (items 10, 18, 20, 23, 28, and 39) were removed due to insufficient consensus or cultural inappropriateness, resulting in the final 43-item version used in this study.

To ensure linguistic and conceptual equivalence, two experienced bilingual translators first translated the original Arabic scale into English. A back-translation procedure was conducted to enhance accuracy, whereby the English version was independently translated back into Arabic and compared with the original to verify correspondence. The finalized instrument was then administered electronically via Google Forms, enabling efficient data collection across both participating institutions.

### Data collection procedures

Data collection was conducted through the electronic administration of the Academic Entitlement Scale. Students were invited to participate voluntarily, with assurances of anonymity and confidentiality. The electronic format facilitated reaching participants across both faculties simultaneously and allowed for efficient data management.

### Data analysis

The data analysis employed a multi-faceted statistical approach to thoroughly examine the scale's psychometric properties. The researchers utilized EFA to investigate the underlying factor structure of the Academic Entitlement Scale within the Egyptian context. This was followed by CFA to verify the identified factor structure. Score reliability assessment was conducted through multiple methods, including internal consistency calculations using Cronbach's alpha and McDonald's omega, and composite reliability estimates. The researchers also examined convergent and discriminant validity through correlational analyses and calculated item-total correlations. All statistical analyses were performed using appropriate statistical software, with a significance level set at *p* < 0.05.

## Results

The psychometric evaluation of the Academic Entitlement Scale within the Egyptian higher education context yielded robust findings regarding its factor structure, reliability, and validity. The analysis proceeded in stages, beginning with EFA to identify the underlying structure, followed by CFA to validate the emergent model, and concluding with comprehensive reliability and validity assessments.

### EFA and scale refinement

The initial 43-item scale was subjected to EFA to examine its latent structure. The Kaiser–Meyer–Olkin (KMO) measure of sampling adequacy was 0.930, well above the recommended threshold of 0.60, indicating that the data were highly suitable for factor analysis. Bartlett's Test of Sphericity was statistically significant (χ^2^ = 13,044.520, df = 1225, *p* < 0.001), confirming that the correlation matrix was appropriate for factorization.

**Fig. 1:**
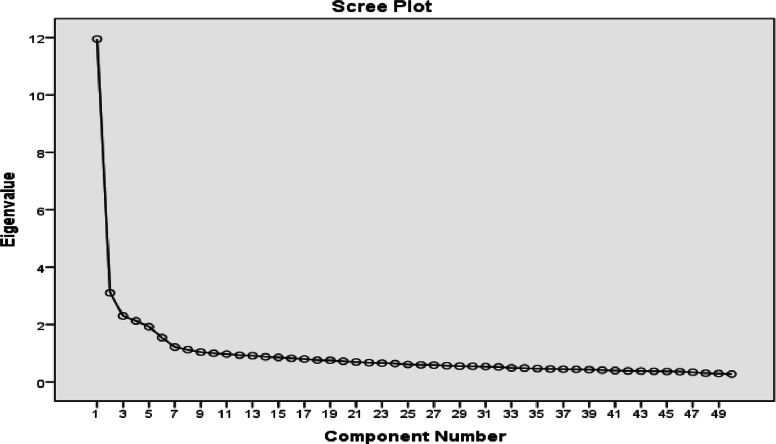
Scree plot of eigenvalues for factor extraction

Multiple criteria were employed to determine the optimal number of factors to retain. The Kaiser criterion (eigenvalues > 1) suggested five factors, which was corroborated by visual inspection of the scree plot (Fig. 1) showing a clear elbow at the fifth factor. Additionally, the five-factor solution demonstrated theoretical coherence and interpretability, aligning with existing literature on academic entitlement dimensions.

### Item reduction and factor structure

Principal component analysis with Varimax rotation and Kaiser normalization was applied with a factor loading cutoff of 0.50 for item retention. The analysis revealed that nine items (1, 2, 4, 8, 11, 16, 21, 32, and 35) failed to load adequately on any factor (loadings < 0.50) and were subsequently removed from the scale. The remaining 34 items demonstrated acceptable factor loadings (all > 0.50) and clear theoretical alignment. The final five-factor solution accounted for 42.819% of the total variance, representing adequate explanatory power for social science research. Table 2 presents the comprehensive variance explained by each factor.

**Table 2:** Total variance explained by the five-factor solution

Factor	Eigenvalues	% of Variance	Cumulative %
1	11.951	23.903	23.903
2	3.103	6.207	30.109
3	2.301	4.603	34.712
4	2.129	4.258	38.970
5	1.924	3.849	42.819

### Factor interpretation and item loadings

The 34-item Academic Entitlement Scale comprised five theoretically coherent factors: (1) Reward for Minimal Effort, capturing expectations of academic success without proportional effort; (2) Control Over Learning Environment, reflecting demands for instructor adaptation to personal preferences; (3) Consumer Mindset, emphasizing transactional views of education; (4) Entitled Expectations, advocating for flexible grading and special accommodations; and (5) Externalized Responsibility, attributing academic struggles to external causes. The complete factor loading matrix is presented in Table 3.

**Table 3:** Factor Loadings for the academic entitlement scale (*N* = 728)

Items	Factor loading				
	**F1**	**F2**	**F3**	**F4**	**F5**
3. If I fail an assignment, it's typically because the instructions were unclear					.508
5. Instructors should adjust their teaching style to match my learning preferences					.629
6. When I struggle academically, it's often due to external circumstances beyond my control					.563
7. The university environment does not provide enough support for my academic success					.511
9. Instructors are responsible for ensuring my academic progress					.544
12. Instructors should provide multiple opportunities to improve my grade				.642	
13. I deserve special consideration in grading due to my unique circumstances				.616	
14. An instructor should modify course requirements if they seem too challenging				.592	
15. I expect detailed, personalized feedback on every assignment				.580	
17. Instructors must always be available to me for consultation				.514	
19. Course policies should be flexible to accommodate my individual needs				.636	
22. My status as a paying student entitles me to preferential treatment			.653		
24. I expect high-quality service in exchange for my tuition fees			.549		
25. Instructors work for me because I'm paying for their services			.597		
26. The university should provide immediate solutions to any academic issues I encounter			.624		
27. My financial contribution should guarantee a certain academic outcome			.618		
29. The university's primary goal is to satisfy my educational demands			.640		
30. My tuition payment guarantees my right to a specific learning experience			.575		
31. I should receive high grades without putting in extensive study time	.704				
33. Minimal effort should be sufficient to pass or excel in courses	.503				
34. I expect recognition and rewards without demonstrating substantial skills	.535				
36. The difficulty of achieving an academic goal should be minimized	.504				
37. I believe talent is more important than hard work in academic success	.552				
38. Studying intensively is unnecessary if I am naturally intelligent	.541				
40. Academic rewards should be granted based on perceived ability, not demonstrated performance	.504				
41. I should have the right to determine course meeting times		.610			
42. Instructors must adapt their teaching methods to my preferred learning style		.705			
43. I expect to choose which course components I want to engage with		.688			
44. The syllabus should be negotiable based on student preferences		.629			
45.I should be able to modify assignment requirements		.629			
46. Classroom policies should be decided democratically by students		.587			
47.I expect to have a say in the evaluation methods used in courses		.650			
48. Technology and teaching tools should be selected based on student input		.588			
49. Course content should be adjusted to match my interests		.615			

F1 = Reward for Minimal Effort; F2 = Control Over Learning Environment; F3 = Consumer Mindset; F4 = Entitled Expectations; F5 = Externalized Responsibility

### Model refinement and final structure

The 34-item structure identified through EFA was subsequently validated using CFA with an independent sample of 599 students. During the CFA process, six items (17, 27, 31, 38, 41, 47) were removed based on modification indices to optimize model fit, resulting in a final 28-item scale. The refined model demonstrated acceptable structural validity within the Egyptian context (Fig. 1).

### Model fit assessment

The CFA results indicated satisfactory model fit across multiple indices. The chi-square to degrees of freedom ratio (CMIN/DF = 2.207) fell within the acceptable range of less than 3.0, suggesting reasonable model fit. The Root Mean Square Residual (RMR = 0.052) was below the recommended threshold of 0.08, indicating minimal discrepancy between observed and predicted covariances. Incremental fit indices demonstrated adequate model performance: Comparative Fit Index (CFI = 0.918), Incremental Fit Index (IFI = 0.919), and Relative Fit Index (RFI = 0.900) all exceeded the 0.90 criterion for acceptable fit. Absolute fit indices also supported model adequacy: Goodness of Fit Index (GFI = 0.913) and Adjusted Goodness of Fit Index (AGFI = 0.904) both surpassed the 0.90 threshold. The Root Mean Square Error of Approximation (RMSEA = 0.045) was well below 0.06, indicating close model fit. Construct score reliability measures showed Average Variance Extracted (AVE = 0.574) above the 0.50 criterion and Composite Reliability (CR = 0.869) exceeding the 0.70 threshold. The comprehensive model fit statistics are summarized in Table 4.

**Table 4:** Model fit indices for the academic entitlement scale

Index	Value	Criterion	Interpretation
CMIN/DF	2.207	< 3.0	Acceptable
RMR	0.052	< 0.08	Good
CFI	0.918	> 0.90	Acceptable
IFI	0.919	> 0.90	Acceptable
RFI	0.900	> 0.90	Acceptable
GFI	0.913	> 0.90	Acceptable
AGFI	0.904	> 0.90	Acceptable
RMSEA	0.045	< 0.06	Good
AVE	0.574	> 0.50	Acceptable
CR	0.869	> 0.70	Good

### Score reliability assessment

Comprehensive score reliability analyses were conducted to evaluate the measurement consistency of the Academic Entitlement Scale. Internal consistency was assessed using multiple coefficients: McDonald's omega (ω), Cronbach's alpha (α), and Guttman's lambda-2 (λ₂). All subscales demonstrated adequate to good score reliability, with McDonald's omega coefficients ranging from 0.714 (Externalized Responsibility) to 0.842 (Control Over Learning Environment). The total scale exhibited excellent score reliability (ω = 0.902). The score reliability coefficients for all subscales and the total scale are presented in Table 5.

**Table 5:** Score reliability coefficients for the academic entitlement subscales and total scale

Factors	McDonald's ω	Cronbach's α	Guttman's λ2
Reward for Minimal Effort	0.747	0.745	0.748
Control Over Learning Environment	0.842	0.841	0.842
Consumer Mindset	0.755	0.752	0.755
Entitled Expectations	0.804	0.803	0.804
Externalized Responsibility	0.714	0.710	0.714
Total	0.902	0.903	0.905

### Test–retest score reliability

Temporal stability was examined through test–retest score reliability with 159 participants over a 17-day interval. All correlations were statistically significant and demonstrated acceptable to good stability: Reward for Minimal Effort (*r* = 0.860, *p* < 0.01), Control Over Learning Environment (*r* = 0.866, *p* < 0.01), Consumer Mindset (*r* = 0.622, *p* < 0.01), Entitled Expectations (*r* = 0.561, *p* < 0.01), Externalized Responsibility (*r* = 0.667, *p* < 0.01), and Total Scale (*r* = 0.870, *p* < 0.01).

### Inter-factor correlations

Examination of inter-factor correlations, based on raw factor scores computed as the mean of items within each subscale, revealed moderate positive associations among all subscales, supporting the multidimensional yet unified nature of academic entitlement. Correlations between factors ranged from 0.366 (Reward for Minimal Effort with Entitled Expectations and Externalized Responsibility) to 0.551 (Consumer Mindset with Entitled Expectations). All factor-total correlations were substantial, ranging from 0.673 to 0.810, indicating that each subscale—represented by the average of its items—contributes meaningfully to the overall construct while maintaining distinctiveness. The complete correlation matrix is presented in Table 6.

**Table 6:** Inter-factor correlations for the academic entitlement scale

Factor	1	2	3	4	5	6
1. Reward for Minimal Effort	1					
2. Control Over Learning Environment	.415^**^	1				
3. Consumer Mindset	.405^**^	.539^**^	1			
4. Entitled Expectations	.366^**^	.525^**^	.551^**^	1		
5. Externalized Responsibility	.366^**^	.401^**^	.381^**^	.449^**^	1	
6. Total	.673^**^	.810^**^	.777^**^	.769^**^	.678^**^	1

** p < *.01*

**Fig. 2:**
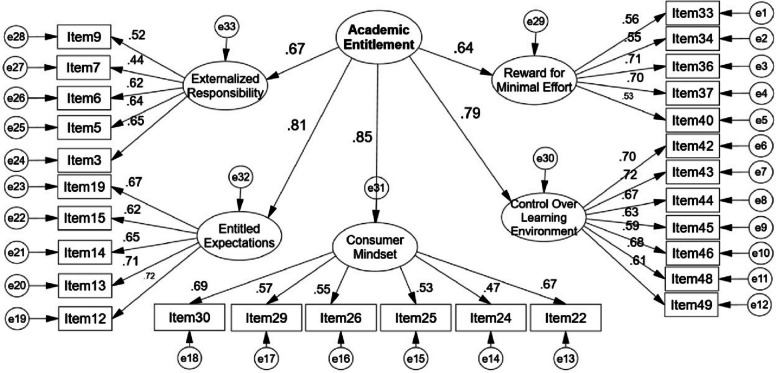
CFA Model for the academic entitlement scale

The psychometric evaluation demonstrates that the 28-item Academic Entitlement Scale possesses satisfactory reliability and validity for measuring academic entitlement among Egyptian university students. The five-factor structure provides a comprehensive framework for understanding the multifaceted nature of academic entitlement within the Egyptian higher education context Fig. 2.

## Discussion

### Factor structure and cross-cultural validity

The current study examined the reliability and validity of the Academic Entitlement Scale within the Egyptian higher education context, contributing to the growing body of research on academic entitlement measurement across diverse cultural settings. The findings support the psychometric robustness of the adapted 28-item scale, which demonstrated a clear five-factor structure, acceptable internal consistency, and good model fit.

The five-factor structure identified—Reward for Minimal Effort, Control Over Learning Environment, Consumer Mindset, Entitled Expectations, and Externalized Responsibility—reflects both consistency and divergence from prior academic entitlement models. The Reward for Minimal Effort and Externalized Responsibility factors closely mirror dimensions found in Jackson et al.'s Academic Entitlement Scale [4] and Chowning and Campbell's two-factor model [8], reinforcing the cross-cultural relevance of these constructs. However, the emergence of Control Over Learning Environment and Consumer Mindset as distinct factors suggests that Egyptian students may place greater emphasis on transactional and instructor-adaptability expectations compared to Western samples. These results align with previous research on academic entitlement while also highlighting unique cultural considerations relevant to Egyptian university students.

The five-factor structure identified in this study—Reward for Minimal Effort, Control Over Learning Environment, Consumer Mindset, Entitled Expectations, and Externalized Responsibility—reflects both consistency and divergence from prior academic entitlement models. The Reward for Minimal Effort and Externalized Responsibility factors closely mirror dimensions found in Jackson et al.'s Academic Entitlement Scale [4] and Chowning and Campbell's two-factor model [8], reinforcing the cross-cultural relevance of these constructs. However, the emergence of Control Over Learning Environment and Consumer Mindset as distinct factors suggests that Egyptian students may place greater emphasis on transactional and instructor-adaptability expectations compared to Western samples. This aligns with research highlighting the influence of consumerist attitudes in higher education [24, 26] but extends it by demonstrating their salience in a non-Western context.

The Entitled Expectations factor, which includes demands for flexible grading and accommodations, resonates with findings from Kopp et al. [29] and Bertl et al. [6], who linked such expectations to narcissistic traits and lower academic integrity. However, the acceptable correlation between this factor and Control Over Learning Environment (*r* = 0.498) suggests that Egyptian students who expect grade leniency also seek influence over instructional methods—a dynamic less emphasized in Western academic entitlement research. This may reflect cultural differences in power distance and student-instructor relationships, where Egyptian students, influenced by hierarchical educational traditions, might simultaneously expect deference from instructors while asserting personal demands.

### Psychometric properties

The scale demonstrated acceptable reliability, with McDonald's omega coefficients ranging from 0.714 (Externalized Responsibility) to 0.842 (Control Over Learning Environment), and excellent overall reliability (ω = 0.902). The scale's convergent validity was supported by composite reliability (CR = 0.869) and average variance extracted (AVE = 0.574), indicating adequate construct measurement. Test–retest reliability over 17 days showed acceptable to good temporal stability, with correlations ranging from 0.561 to 0.870.

The moderate inter-factor correlations (*r* = 0.366–0.551) suggest that while the subscales are distinct, they collectively represent the broader academic entitlement construct. The weakest correlation between Reward for Minimal Effort and both Entitled Expectations and Externalized Responsibility (*r* = 0.366) underscores their conceptual independence, supporting the multidimensional nature of academic entitlement.

### Cultural considerations in Egyptian context

The adaptation of the Academic Entitlement Scale to Egyptian higher education revealed several culturally specific nuances. First, the prominence of the Control Over Learning Environment factor may reflect the centralized and instructor-dominated nature of Egyptian education, where students historically have had limited autonomy in shaping their learning experiences. The demand for greater control could thus represent a reaction to traditional pedagogical structures, akin to findings in Turkish adaptations [30, 31].

Second, the Consumer Mindset factor’s acceptable psychometric performance highlights the growing influence of neoliberal education policies in Egypt, where rising tuition costs and privatization have fostered student perceptions of higher education as a service-for-payment exchange. This aligns with global trends [26] but may be particularly pronounced in Egypt due to economic pressures and high youth unemployment, which intensify the perceived ROI of a degree.

### Practical implications

The validated scale provides a valuable tool for assessing academic entitlement in Egyptian and similar Arab educational contexts. Its multidimensional structure allows researchers to explore nuanced relationships between academic entitlement dimensions and academic outcomes. Universities could design interventions addressing specific factors, such as orientation programs emphasizing effort-based achievement to counter Reward for Minimal Effort expectations.

### Study limitations

The cross-sectional design represents a significant limitation that affects the scale's application readiness. This design precludes understanding of academic entitlement's developmental trajectory, stability over time, or causal relationships with academic outcomes. The inability to establish temporal precedence limits conclusions about whether academic entitlement leads to poor academic behaviors or vice versa, restricting the scale's immediate utility for predictive purposes.

A critical limitation is the lack of predictive validity assessment. The study did not examine whether academic entitlement scores predict actual behavioral outcomes such as academic performance, dishonesty, or student-faculty conflicts. This absence of predictive validity testing means the scale's practical utility for identifying at-risk students or evaluating intervention effectiveness remains unestablished and should be a primary focus in future research.

The sample was limited to Al-Azhar University students, which may affect generalizability to other Egyptian institutions with different educational philosophies or student populations. The convenience sampling approach may have introduced selection bias, potentially limiting the representativeness of findings across diverse Egyptian higher education contexts.

### Future research directions

Future research should prioritize longitudinal designs to establish predictive validity and examine academic entitlement developmental patterns. Cross-cultural comparisons with other Arab universities would enhance generalizability, while linking academic entitlement scores to objective academic and behavioral outcomes would establish practical utility. Investigation of intervention efficacy targeting specific academic entitlement dimensions represents another crucial research avenue.

## Conclusion

The Academic Entitlement Scale demonstrates acceptable psychometric properties in the Egyptian higher education context, capturing five distinct yet interrelated dimensions of academic entitlement. Its reliability, validity, and cultural appropriateness make it a valuable tool for researchers and educators seeking to understand and address entitlement behaviors in Arab universities. This study contributes to a more globally inclusive understanding of academic entitlement and its implications for student success and institutional quality by bridging the gap between Western academic entitlement research and non-Western educational settings. Future research should build on these findings to explore cross-cultural comparisons and the efficacy of targeted interventions to foster student accountability and realistic academic expectations.

## Data Availability

The datasets generated and analyzed during the current study are available from the corresponding author upon reasonable request.

## References

[1] Seipel S, Brooks N. Academic entitlement beliefs of information systems students: a comparison with other business majors and an exploration of key demographic variables and outcomes. Inf Syst Educ J. 2020;18(4):46–58.

[2] Wolthuis S, Slade C. Liberating legacy system data with Rails, intelligent use of conflict data with automated class scheduling tools. Inf Syst Educ J. 2020;18(4):12–21.

[3] Seipel S, Brooks N, Baran B, Chao JY. Academic entitlement in graduate students: scale development and validation. J Sch Teach Learn. 2020;20(3):88–110.

[4] Jackson D, Frey M, McLellan C, Rauti C, Lamborn P, Singleton-Jackson J. I deserve more A’s: a report on the development of a measure of academic entitlement. PLoS One. 2020. 10.1371/journal.pone.0239721. 32997723 10.1371/journal.pone.0239721 PMC7526903

[5] Fromuth M, Bass J, Kelly D, Davis T, Chan K. Academic entitlement: its relationship with academic behaviors and attitudes. Soc Psychol Educ. 2019;22:1153–67. 10.1007/s11218-019-09517-2.

[6] Bertl B, Andrzejewski D, Hyland L, Shrivastava A, Russell D, Pietschnig J. My grade, my right: linking academic entitlement to academic performance. Soc Psychol Educ. 2019;22:775–93. 10.1007/s11218-019-09509-2.

[7] Turner L, McCormick W. Academic entitlement: relations to perceptions of parental warmth and psychological control. Educ Psychol. 2018;38(2):248–60. 10.1080/01443410.2017.1328487.

[8] Chowning K, Campbell NJ. Development and validation of a measure of academic entitlement: individual differences in students’ externalized responsibility and entitled expectations. J Educ Psychol. 2009;101(4):982–97. 10.1037/a0016351.

[9] Bonaccio S, Reeve C, Lyerly J. Academic entitlement: its personality and general mental ability correlates, and academic consequences. Pers Individ Differ. 2016;102:211–6. 10.1016/J.PAID.2016.07.012.

[10] Reinhardt J. Conceptualizing Academic Entitlement: What are we Measuring? [Master thesis]. University of Windsor; 2012.

[11] Badawy ME, Soliman T, Nemt-Allah MA. Navigating adversity: the relationship between academic entitlement and resilience in male undergraduates. Int J Eval Res Edu. 2025;14(4):3042–50. 10.11591/ijere.v14i4.32845.

[12] Cook A, Egan H, Wood J, Mantzios M. Examining the relationship of depression and anxiety to academic entitlement, and the potential mediating role of mindfulness. J Further High Educ. 2023;47(9):1211–20. 10.1080/0309877X.2023.2241382.

[13] Taylor J, Bailey S, Barber L. Academic entitlement and counterproductive research behavior. Pers Individ Differ. 2015;85:13–8. 10.1016/J.PAID.2015.04.024.

[14] Abdellatif MS. Moral intelligence and its relationship to academic entitlement and academic performance of secondary school students. Eur J Educ Res. 2022;11(4):2291–301. 10.12973/eu-jer.11.4.2291.

[15] Reysen R, Degges-White S, Reysen M. Exploring the interrelationships among academic entitlement, academic performance, and satisfaction with life in a college student sample. J Coll Stud Retent-R. 2020;22(2):186–204. 10.1177/1521025117735292.

[16] Shafait Z, Sahibzada U. I deserve better grades." Compliance-gaining perspective of dark triad traits, power distance and academic entitlement in Chinese higher education. Kybernetes. 2024;54(4):2224–44. 10.1108/k-08-2023-1454.

[17] Turnipseed D, Cohen S. Academic entitlement and socially aversive personalities: Does the Dark Triad predict academic entitlement? Personality Individ Differ. 2015;82:72–5. 10.1016/J.PAID.2015.03.003.

[18] Peñero R, Asilom I, Bautista I, Burgos S, Clemente J, Rodriguez R, et al. Academic entitlement among Filipino college students: exploring the influence of narcissism, locus of control, and family functioning. Acad Lasalliana J Educ Human. 2024;5(2):11–22. 10.55902/nvnf2161.

[19] Greenberger E, Lessard J, Chen C, Farruggia S. Self-Entitled College Students: Contributions of Personality, Parenting, and Motivational Factors. J Youth Adolesc. 2008;37:1193–204. 10.1007/S10964-008-9284-9.

[20] McLellan C, Jackson D. Personality, self-regulated learning, and academic entitlement. Soc Psychol Educ. 2017;20:159–78. 10.1007/S11218-016-9357-7.

[21] Huang S, Kuo B. Demographic, psychosocial, and cultural predictors of entitlement in a multiethnic Canadian undergraduate sample. Soc Psychol Educ. 2020;23:523–35. 10.1007/s11218-020-09547-1.

[22] Abdellatif M, Elagamy AA, Abdelgawad BIA. The effectiveness of a moral intelligence training program in reducing secondary school students’ academic entitlement. Res J Adv Human. 2024;5(2):101–18. 10.58256/n2gmwj17.

[23] Kinne B, Goehring M, Williams B. Academic Entitlement and Its Potential Educational Consequences: A Scoping Review. J Phys Therap Educ. 2022;36(2):115–21. 10.1097/JTE.0000000000000231.

[24] Cain J, Romanelli F, Smith KM. Academic entitlement in pharmacy education. Am J Pharm Educ. 2012;76(10):189. 10.5688/ajpe7610189. 23275654 10.5688/ajpe7610189 PMC3530051

[25] Stroebe W. Student evaluations of teaching encourages poor teaching and contributes to grade inflation: a theoretical and empirical analysis. Basic Appl Soc Psychol. 2020;42(4):276–94. 10.1080/01973533.2020.1756817.

[26] Berezvai Z, Lukáts GD, Molontay R. Can professors buy better evaluation with lenient grading? The effect of grade inflation on student evaluation of teaching. Assess Eval High Educ. 2020;46(5):793–808. 10.1080/02602938.2020.1821866.

[27] Malone DE Jr, Johnson BC. Pressure to Please: Adjunct Faculty Experiences with Grade Inflation. J High Educ Policy Leader Stud. 2023;4(3):75–95. 10.61186/johepal.4.3.75

[28] Schutz KR, Drake BM, Lessner J, Hughes GF. A comparison of community college full-time and adjunct faculties’ perceptions of factors associated with grade inflation. J Contin High Educ. 2015;63(3):180–92. 10.1080/07377363.2015.1085951.

[29] Jiang L, Tripp TM, Hong PY. College instruction is not so stress free after all: a qualitative and quantitative study of academic entitlement, uncivil behaviors, and instructor strain and burnout. Stress Health. 2017;33(5):578–89. 10.1002/smi.2742. 28105661 10.1002/smi.2742

[30] Knepp KA, Knepp MM. Academic entitlement decreases engagement in and out of the classroom and increases classroom incivility attitudes. Soc Psychol Educ. 2022;25(5):1113–34. 10.1007/s11218-022-09716-4. 35873869 10.1007/s11218-022-09716-4 PMC9289648

[31] Kopp JP, Finney SJ. Linking academic entitlement and student incivility using latent means modeling. J Exp Educ. 2013;81(3):322–36. 10.1080/00220973.2012.727887.

[32] Small SP, Cashin G, English D, Moran G. It is essentially about treating each other well”: insights from faculty on incivility in nursing education. Can J Nurs Res. 2023;56(1):81–94. 10.1177/08445621231204985. 37788344 10.1177/08445621231204985 PMC10804871

[33] Zhu L, Anagondahalli D. Effects of academic entitlement on conflict management: implications of a consumer culture for the student-teacher relationship. Commun Rep. 2017;30(1):14–25. 10.1080/08934215.2016.1225223.

[34] Heffernan K, Gates T. Perceptions of teaching staff in human services about academic entitlement. J Appl Res High Educ. 2018;10(4):469–77. 10.1108/JARHE-11-2017-0143.

[35] Bluestein S. Connecting student-faculty interaction to academic dishonesty. Community Coll J Res Pract. 2015;39(2):179–91. 10.1080/10668926.2013.848176.

[36] Zhu L, Anagondahalli D. Predicting student satisfaction: the role of academic entitlement and nonverbal immediacy. Commun Rep. 2018;31(1):41–52. 10.1080/08934215.2017.1364777.

[37] Kopp J, Zinn T, Finney S, Jurich D. The development and evaluation of the academic entitlement questionnaire. Meas Eval Couns Dev. 2011;44(2):105–29. 10.1177/0748175611400292.

[38] Kurtyılmaz Y. Adaptation of Academic Entitlement Questionnaire. Anadolu J Educ Sci Int. 2019;9(2):314–351. 10.18039/AJESI.577234.

[39] Kantar A, Cevheroglu B, Bakir A, Çi̇çek S. Examining the validity and reliability of the academic entitlement scale in Turkish culture. Int J Asses Tool Educ. 2024;11(1):195–212. 10.21449/ijate.1364169.

